# Reward Sensitivity and Waiting Impulsivity: Shift towards Reward Valuation away from Action Control

**DOI:** 10.1093/ijnp/pyx072

**Published:** 2017-08-08

**Authors:** Daisy J Mechelmans, Daniela Strelchuk, Nuria Doñamayor, Paula Banca, Trevor W Robbins, Kwangyeol Baek, Valerie Voon

**Affiliations:** Department of Psychiatry, University of Cambridge, Addenbrooke’s Hospital, Cambridge, United Kingdom (Ms Mechelmans, Ms Strelchuk, Dr Donamayor-Alonso, Dr Banca, Dr Baek, and Dr Voon); KU Leuven – University of Leuven, Department of Neurosciences, Leuven, Belgium (Ms Mechelmans); Behavioural and Clinical Neurosciences Institute, University of Cambridge, Cambridge, United Kingdom (Drs Robbins and Voon); Cambridgeshire and Peterborough NHS Foundation Trust, Cambridge, United Kingdom (Dr Voon); NIHR Cambridge Biomedical Research Centre, Cambridge, United Kingdom (Dr Voon)

**Keywords:** impulsivity, reward, orbitofrontal cortex, supplementary motor area, monetary incentive delay

## Abstract

**Background:**

Impulsivity and reward expectancy are commonly interrelated. Waiting impulsivity, measured using the rodent 5-Choice Serial Reaction Time task, predicts compulsive cocaine seeking and sign (or cue) tracking. Here, we assess human waiting impulsivity using a novel translational task, the 4-Choice Serial Reaction Time task, and the relationship with reward cues.

**Methods:**

Healthy volunteers (n=29) performed the monetary incentive delay task as a functional MRI study where subjects observe a cue predicting reward (cue) and wait to respond for high (£5), low (£1), or no reward. Waiting impulsivity was tested with the 4-Choice Serial Reaction Time task.

**Results:**

For high reward prospects (£5, no reward), greater waiting impulsivity on the 4-CSRT correlated with greater medial orbitofrontal cortex and lower supplementary motor area activity to cues. In response to high reward cues, greater waiting impulsivity was associated with greater subthalamic nucleus connectivity with orbitofrontal cortex and greater subgenual cingulate connectivity with anterior insula, but decreased connectivity with regions implicated in action selection and preparation.

**Conclusion:**

These findings highlight a shift towards regions implicated in reward valuation and a shift towards compulsivity away from higher level motor preparation and action selection and response. We highlight the role of reward sensitivity and impulsivity, mechanisms potentially linking human waiting impulsivity with incentive approach and compulsivity, theories highly relevant to disorders of addiction.

**Significance Statement**

Impulsivity and reward sensitivity are commonly related. We show in healthy controls with high waiting impulsivity that seeing a cue predicting high reward is associated with a shift towards brain regions involved in linking reward value and choices away from higher order motor control. Reward sensitivity may link waiting impulsivity with habit and incentive motivation, theories relevant to addiction.

## Introduction

Impulsivity and reward expectancy are commonly interrelated. Waiting impulsivity, also known as premature responding, has been identified as both a predictor and consequence of substance use disorders in rodent studies (Robbins, 2002; Voon and Dalley, 2015). In preclinical studies, premature responding is studied using the 5-choice serial reaction time task (5-CSRT) (Robbins, 2002), a visuospatial task in which rodents learn to respond to a visual cue predicting reward. High waiting impulsivity in rodents predicts the transition to compulsive cocaine-seeking behaviors, enhanced acquisition of nicotine self-administration, and alcohol preference in mice (Belin et al., 2008; Diergaarde et al., 2008; Voon and Dalley, 2015). Greater rodent sign-tracking, or approach behaviors towards the incentive cue, is also associated with high waiting impulsivity (Lovic et al., 2011). This potential relationship between waiting impulsivity and habit and incentive motivation is highly relevant for individual differences in impulsivity and reward sensitivity and underpins key conceptual theories underlying addictions. Here we examine the relationship between waiting impulsivity and reward sensitivity in humans.

Impulsivity is the tendency to react without adequate forethought and control, irrespective of negative consequences (Moeller et al., 2001). Impulsivity is a multidimensional construct, of which waiting impulsivity is a subtype (Dalley et al., 2011). Other forms include motor (response inhibition) and decisional (delay discounting and reflection impulsivity) forms (Voon and Dalley, 2015). Using a novel translational human analogue of the rodent 5-CSRT, the 4-choice serial reaction time task (4-CSRT), individuals with disorders of addiction (alcohol and methamphetamine dependent and current nicotine and cannabis users) were shown to have elevated premature responding (Voon et al., 2014). Binge drinkers at elevated risk of alcohol use disorders also showed elevated waiting impulsivity, suggesting a potential role for waiting impulsivity as a risk predictor (Sanchez-Roige et al., 2014; Morris et al., 2016a). In rodents, the neural network underlying waiting impulsivity in the rodent 5-CSRT has been extensively mapped and documented. Special interest falls on the infralimbic cortex, equivalent to the human subgenual anterior cingulate (sgACC) (Voon and Dalley, 2015) and the subthalamic nucleus (STN). Lesions of the infralimbic cortex or STN (Baunez and Robbins, 1997) enhance premature responding. In high impulsive rodents, the nucleus accumbens is associated with lower D2,3 receptor density (Dalley et al., 2007) and lower left-sided volume. In humans, waiting impulsivity was associated with lower resting state functional connectivity of sgACC, ventral striatal and STN network (Morris et al., 2016a), regions implicated in lesion and pharmacological studies in rodents (Voon and Dalley, 2015). STN connectivity, particularly to the sgACC, further predicted alcohol misuse in binge drinkers and alcohol use disorders (Morris et al., 2016a). As a relay center, the STN has a crucial role in inhibitory function and has been implicated in impulse control. The STN is an important mediator for the switch from automatic behavior to controlled processing, including to inhibit behavior.

A well-validated paradigm for investigating neural activity in the anticipation of reward is the monetary incentive delay task (MID) (Knutson et al., 2000). Subjects are shown a cue predicting the magnitude of the reward outcome and then are required to wait for a target prior to responding as quickly as possible. A meta-analysis of the MID task in healthy controls showed greater ventral striatal activity during reward anticipation (Knutson and Greer, 2008) with greater medial orbitofrontal cortex (mOFC) during reward receipt, and particularly with receipt of high magnitude rewards (Diekhof et al., 2012). The MID task has been extensively investigated in disorders of addiction (Balodis and Potenza, 2015). The relationship between impulsivity and neural activity in the MID task has thus far focused on self-reported impulsivity questionnaires and delay discounting, demonstrating a negative relationship between ventral striatal neural activity and impulsivity (Beck et al., 2009; Andrews et al., 2011; Peters et al., 2011; Balodis et al., 2012; Benningfield et al., 2014), consistent with the rodent literature (Caprioli et al., 2014). Here, we ask how waiting impulsivity is related to reward predicting cues in the MID task when tested in the same individuals. We assess both low and high monetary reward magnitudes (£1 and £5) and hypothesize that waiting impulsivity, similar to self-reported impulsivity and delay discounting, will be associated with lower ventral striatal and mOFC activity to high magnitude rewards.

## Materials and Methods

### Participants

Healthy volunteers were recruited from the Behavioural and Clinical Neuroscience Institute healthy volunteer list and community-based advertisements. Exclusion criteria included the presence of a major psychiatric disorder or substance use disorder, being under 18 years of age, current major medical or neurological illness, or use of psychoactive medications. Participants completed the National Adult Reading Test to determine verbal IQ (Nelson, 1982) and the Beck Depression Inventory (Beck et al., 1961) and Spielberger Trait Anxiety Inventory. Participants were reimbursed for their time and written informed consent was obtained. The study was approved by the University of Cambridge Research Ethics Committee.

Twenty-nine healthy volunteers (16 females and 13 males, mean age 23.65 years [SD 4.44], verbal IQ 108.84 [SD 8.75]) completed the MID and the 4CSRT. Participants scored 7.93 (SD 6.08) on the BDI and 40.48 (SD 10.88) on the STAI.

Participants performed the MID task in the scanner and were tested on the 4-CSRT outside of the scanner.

### Monetary Incentive Delay Task

**Figure 1.**
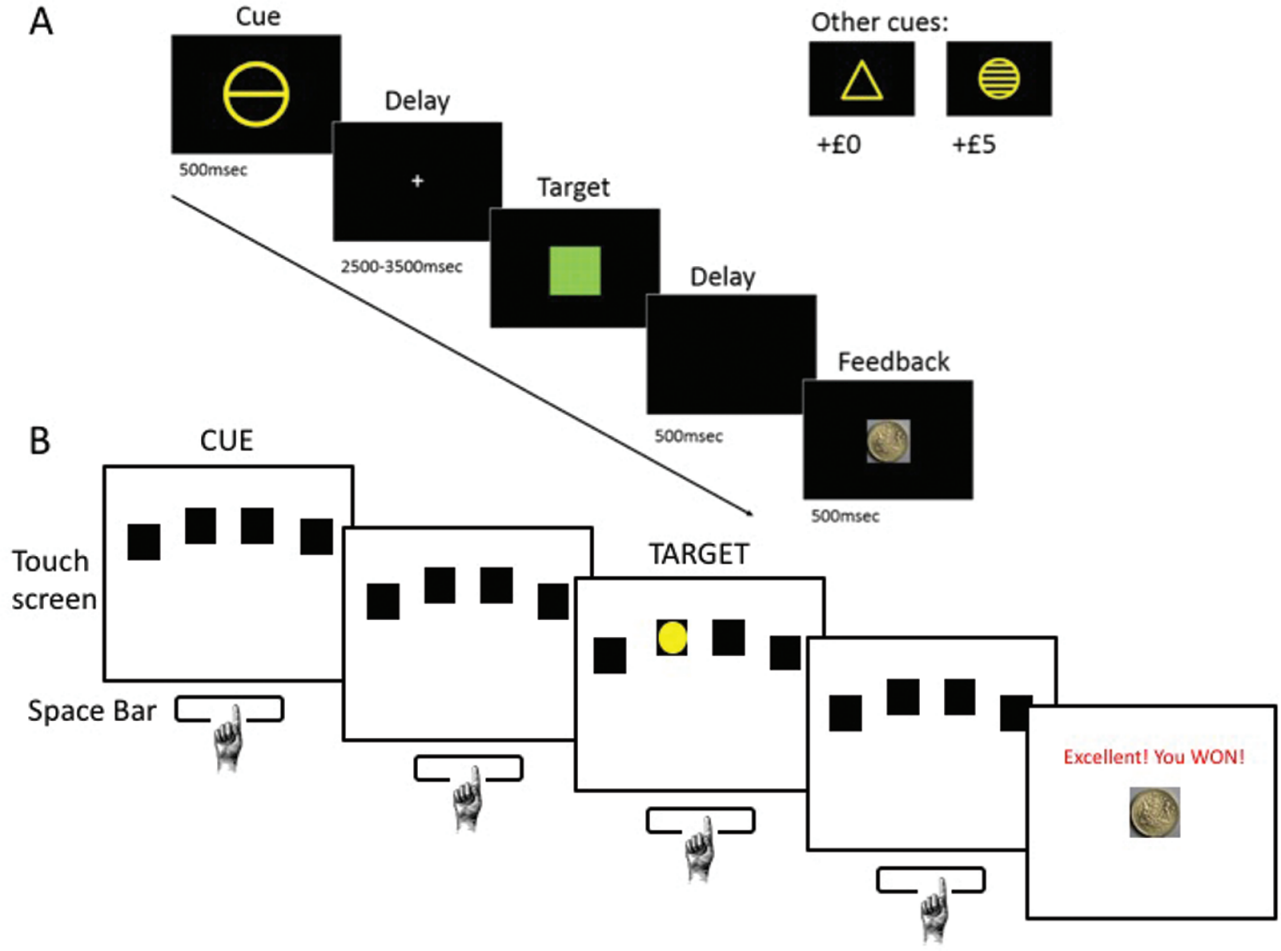
Imaging and behavioral task. (A) The Monetary Incentive Delay (MID) imaging task adapted from Knutson et al. (2000). Two cues (circles with 1 and 5 lines) predicted £1 and £5 reward, respectively, and 1 cue (triangle) predicted no reward (£0). A timely response button during the target presentation led to the receipt of the reward and a decrease in the target duration. A late response resulted in an increase in the target duration. (B) The 4-Choice Serial Reaction Time task (4-CSRT) was tested offline. Participants were seated in front of a touch screen with 4 boxes and instructed to press and hold the space bar, which indicated the cue-onset time. At the occurrence of a green dot, participants released the space bar and touched the box where the green dot had appeared. Participants were instructed to be as fast as possible. The number of the premature releases before the occurrence of the target was the primary outcome of the task.

We used the MID task to examine neural responses during anticipation of reward (£5, £1, or £0) (Knutson et al., 2000). Participants were first shown 1 of 3 yellow figures (Figure 1A) indicating they could either win £5, £1, or nothing (cue phase, 500 milliseconds) followed by a fixation cross (response anticipation phase, variable delay 2500 to 3500 milliseconds). The target (green square) was initially shown for 500 milliseconds with the target duration changing depending on the rapidity of responding. If they responded within the time frame of the green square target (500 milliseconds), they won the corresponding amount and the target duration shortened by -50 milliseconds. If they failed to respond within the time frame of the target duration, they won nothing and the target duration increased by 50 milliseconds. Thus, participants had to respond as fast as possible to gain money and were told they would receive a monetary proportion of their score after the experiment. The duration of the target and response was independently tracked and adjusted for each of the 3 conditions. Following the target was a delay of 500 milliseconds prior to the feedback (500 milliseconds). The feedback display for the control, £1 reward, and £5 reward conditions respectively showed a grey square, a £1 coin, or a £5 note. Incorrect responses and no responses were followed by a black screen. Between trials, a jittered screen instructed the participant the next cue was about to be presented (500–2500 milliseconds). The experiment consisted of 35 control, 35 £1 reward, and 35 £5 reward cues in random order. Outcome variables for the MID were the reaction time of the final 5 correct trials and proportion of correct trials.

### 4-CSRT

The 4-CSRT has been described extensively (Voon et al., 2014) and will be only briefly described here (Figure 1B). Participants sat in front of a touch screen displaying 4 boxes and held down the space bar with their dominant index finger on the keyboard, indicating cue-onset time. A visual cue (green dot) randomly appeared in 1 of the 4 boxes. Subjects were required to release the space bar and to touch the box on the screen in which the target appeared. The primary outcome measure was the number of premature releases (i.e., release of the space bar prior to onset of the visual cue). Following a premature response, subjects were required to complete the trial by touching the screen, and a feedback display presented “keep going” without receiving a monetary reward. The task was divided into 2 baseline blocks without monetary reward to individualize monetary feedback based on the individual’s mean fastest reaction time and SD and 4 test blocks. The 4 test blocks with monetary reward were optimized with long and short target durations, fixed and variable time intervals, and the introduction of distractor nontargets to increase premature responding. The task lasted 20 minutes in total and was programmed in Visual Basic with Visual Studio 2005.

### Imaging Parameters

Images were acquired with a Siemens 3T Tim Trio scanner using a 32-channel head coil at the Wolfson Brain Imaging Centre at the University of Cambridge. For anatomical reference, a T1-weighted magnetization prepared rapid gradient echo was acquired (FOV 240 x 256 x 176 mm, 1-mm-in-plane resolution, inversion time [TI] = 900 milliseconds, TR = 2300 milliseconds; TE = 2.98 milliseconds; flip angle = 9°; voxel size = 1 x 1 x 1 mm). For the acquisition of the functional images, the following parameters were used: TR = 2.32 seconds, TE = 30 milliseconds, flip angle = 78°, matrix = 64 x 64, voxel size = 3 x 3 x 3 mm^3^, a 25% gap between slices (0.75 mm).

### Analysis

Functional magnetic resonance data were analysed using Statistical Parametric Mapping 8 (Wellcome Trust Centre for Neuroimaging, University College London, UK, www.fil.ion.ucl.ac.uk/spm). After slice time correction, a mean image for all functional scans was generated for each subject, to which individual volumes were spatially realigned by rigid body transformation. Movement parameters were included in the realignment algorithm. Unwarping was performed during realignment to correct for dynamic motion-distortion interaction artefacts. The T1-weighted structural image was co-registered with the mean image of the functional volumes and was segmented into grey and white matter images. The grey matter image was normalized to the a priori grey matter template produced at the Montreal Neurological Institute (MNI). The normalization parameters were then applied to the functional images to ensure an anatomically informed normalization. The resulting images were subsampled into a resolution of 2 x 2 x 2 mm in MNI space. A Gaussian filter of 8 mm Full Width at Half Maximum (FWHM) was then applied to smooth the data spatially to take into account the anatomical variability between participants and to satisfy the assumptions of Gaussian random field theory for controlling multiple comparisons in the analysis. Individual data were inspected for head motion artefact >5 mm.

### Data Processing

Behavioral data from the 4CSRT and from the MID task were inspected for outliers and normality of distribution (Shapiro-Wilkes *P*>.05). Outliers were removed from analysis (>3 SD from group mean). As the outcomes from the MID task were not normally distributed, the relationship between reward magnitude and percentage correct and RT were compared using nonparametric related samples Friedman’s 2-way ANOVA by ranks. On an exploratory basis, the relationship between premature responses and neural activity with these variables was assessed using Spearman’s rank correlation coefficient.

For the imaging analyses, at the first level, onset and duration were modelled for cue (duration: 0.5 seconds), anticipation (duration: 2.5–3.5 seconds), response, and outcome (duration: 0.5 seconds). Second level analyses were conducted using general linear modelling to assess the effects of reward magnitude in the contrasts of £1-neutral and £5-neutral in the reward cue phase with outcomes of £1, £5, and no win assessed on an exploratory basis. The primary hypothesis was assessed using a regression analysis focusing on the outcome of premature responding from the 4CSRT examined as a regressor for both the £5-neutral cue and £1-neutral cue with age and gender as covariates of no interest. Whole-brain family-wise error (FWE) cluster level corrected *P*<.05 was considered significant. As the ventral striatum and mOFC were regions identified in meta-analyses of the MID task and were a priori hypothesized to be related to impulsivity, small volume corrected region of interest (ROI) corrected *P*<.025 (Bonferroni correction for 2 ROIs) was considered significant. The ventral striatal anatomical ROI, previously used in other studies (Murray et al., 2008), had been hand drawn in MRIcro following the definition of ventral striatum by Martinez et al. The mOFC ROI was based on previously defined ROIs from our previous studies (Morris et al., 2016b). For the OFC, the dorsal extent was defined by the axial slice showing the disappearance of the olfactory sulcus, and the medial and lateral OFC were distinguished by the crown of the gyrus rectus. The mOFC ROI consisted of the combination of 2 boxes (6 x 26 x 4 mm) and centered on coordinates (±6, 36, -22).

On an exploratory level, psychophysiological interaction analyses comparing high and low reward cues were conducted with the bilateral mOFC, a critical region identified in the analysis, and bilateral seeds in the STN, VS, and SgAcc, regions identified in our previous study of human neural correlates of the 4-CSRT (Morris et al., 2016a) and on known rodent lesion studies (Voon and Dalley, 2015) with whole brain cluster-level corrected FWE *P*<.0125 considered significant (Bonferroni corrected for 4 seeds).

## Results

### Behavioral Results

On average, participants made 5.55 premature responses in the 4CSRT (min = 0; max = 20; mean = 5.55; SD = 4.71). Two outliers in the 4CSRT (>3 SD from the mean) were removed from further analysis. In the MID task, as a function of reward magnitude, there were differences in accuracy (control: 56.35% [SD 1.97]; £1: 57.33% [SD 1.97]; £5: 57.11 [SD 1.99], *P*=.045) and RT (control: 217.03 [SD 37.01]; £1: 215.96 [SD 41.69]; £5: 206.65 [SD 36.14], *P*=.014). These findings highlight the sensitivity of the MID task to reward prospect. There was no relationship between premature responses on the 4CSRT and these variables (*P*>.05).

### Imaging Results

**Figure 2.**
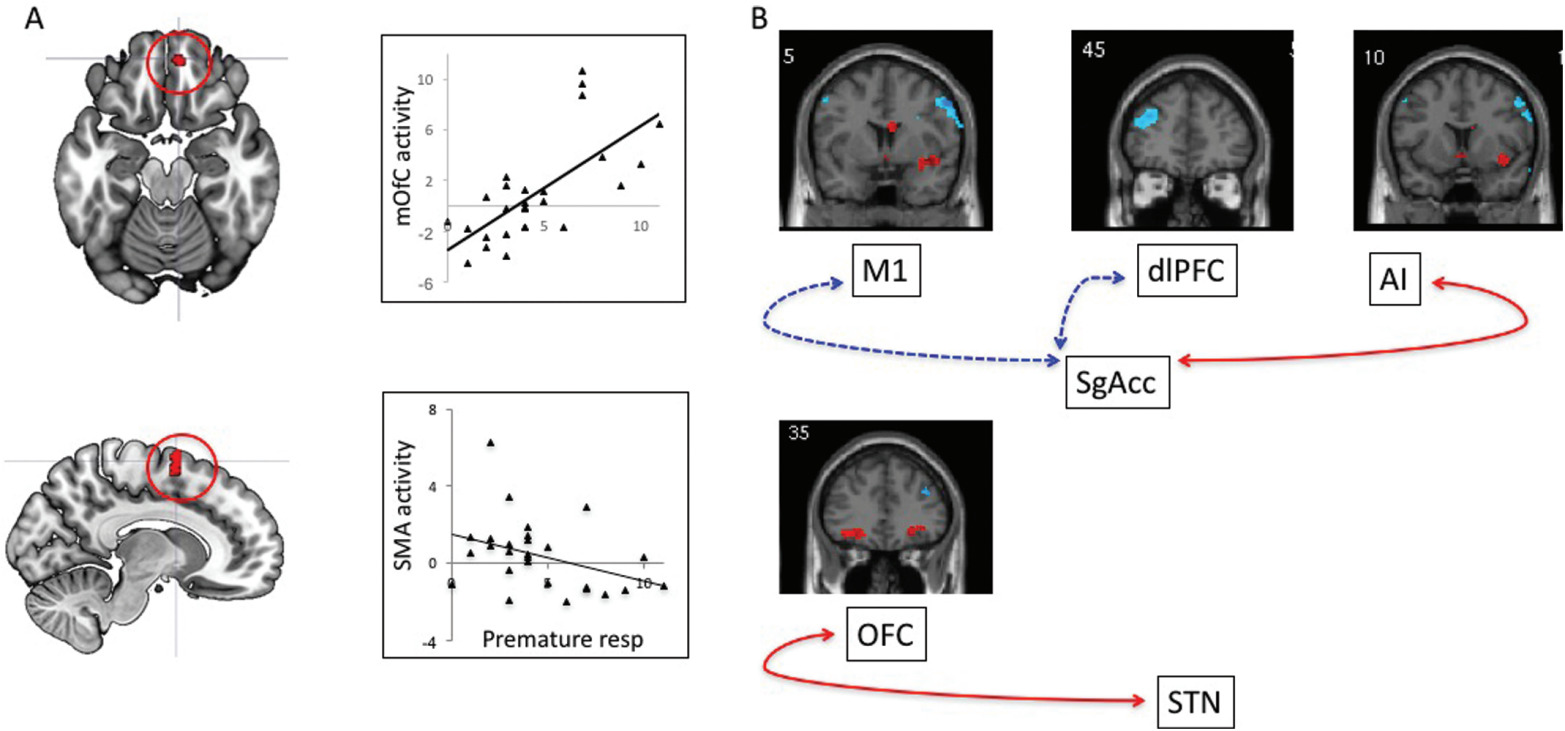
Neural correlates of waiting impulsivity in the monetary incentive delay task. (A) Neural correlates of waiting impulsivity as a regressor in the monetary incentive delay task as a function of high reward cue (top: £5-neutral). Top: The image and graphs show that high waiting impulsivity was positively correlated with medial orbitofrontal cortex (mOFC) (top image and graph) and negatively correlated with supplementary motor area (SMA) activity (bottom image and graph) as a function of high reward cues. (B) Psychophysiological interaction analysis of £5-neutral cues showed that waiting impulsivity was correlated with greater functional connectivity between the OFC and subthalamic nucleus (STN) seed and greater connectivity between subgenual cingulate (SgAcc) seed and anterior insula (AI) and lower connectivity between SgAcc seed and dorsolateral prefrontal cortex (dlPFC) and somatomotor cortex (M1). Red lines indicate greater connectivity, blue dashed lines indicate lower connectivity.

The following describes the primary hypothesis of the regressor of waiting impulsivity as measured using the 4-CSRT in the cue phase of the MID task. During the cue phase for the £5-neutral contrast, waiting impulsivity as measured using the 4-CSRT was negatively correlated with supplementary motor area (SMA) activity (peak voxel x, y, and z in MNI coordinates: 6, 6, and 74 mm, respectively; Z = 4.13, cluster corrected FWE *P*=.018) (Figure 2A). The ROI analysis also showed that waiting impulsivity as measured using the 4CSRT was positively correlated with bilateral mOFC activity for the £5-neutral contrast (peak voxel = 6, 50, and -20 mm; Z = 3.94, small volume corrected ROI *P*=.022). There were no significant correlations with the ventral striatal ROI. The £1-neutral cue was not significantly correlated with waiting impulsivity. There were no significant correlations between waiting impulsivity and the outcome phase. There was no relationship between waiting impulsivity measured on the 4-CSRT and behavioral measures of the MID task (RT and proportion correct) (*P*>.05).

We further assessed the £5-neutral cue phase without the regressor reported here as FWE cluster corrected *P*<.05. The £5-neutral cue contrast showed bilateral activation in the ventral striatum (peak voxel reported with MNI coordinates in mm = -10, 8, and 0; Z = 4.22), supplementary motor area (peak voxel = 6, 2, and 76; Z = 4.45), substantia nigra (peak voxel = 8, -16, and -10; Z = 4.27), thalamus (peak voxel = -2, -16, and 10, Z = 3.90), and bilateral anterior insula (L peak voxel = -36, 26, and 0; Z = 5.36; R peak voxel = 36, 24, and -8; Z = 5.15).

We then assessed psychophysiological interactions focusing on bilateral mOFC, STN, VS, and SgAcc. At baseline, without the regressor of impulsivity, the seeds did not show any significant functional connectivity as a function of the £5-neutral contrast during the cue phase. However, high impulsivity during the £5-neutral cue phase was associated with greater connectivity between bilateral STN and left OFC (peak voxel = -26, 36, and -12; cluster size =244, Z=3.85, whole brain cluster level FWE corrected *P*=.007) and greater connectivity between bilateral SgAcc and right insula (peak voxel = 40, 6, and -10; cluster size =457, Z=4.35, whole brain cluster level FWE *P*<.001) and lower connectivity with left dorsolateral prefrontal cortex (dlPFC) (peak voxel = -40, 52, and 20; cluster size=639, Z=4.40, whole brain cluster level FWE *P*<.001) and right motor cortex (peak voxel = 56, 8, and 44; cluster size=238, Z=4.25, whole brain cluster level FWE *P*=.008) (Figure 2B).

## Discussion

We assessed how the ability to wait before responding on the 4-CSRT is associated with reward expectancy in healthy volunteers. In response to high magnitude reward cues, elevated waiting impulsivity on the 4-CSRT was associated with greater mOFC activity and lower SMA activity. Furthermore, high waiting impulsivity in response to high reward cues showed greater connectivity between STN and left mOFC and greater connectivity between SgAcc and right insula and lower connectivity between SgAcc and left dlPFC and right motor cortex.

**Figure 3.**
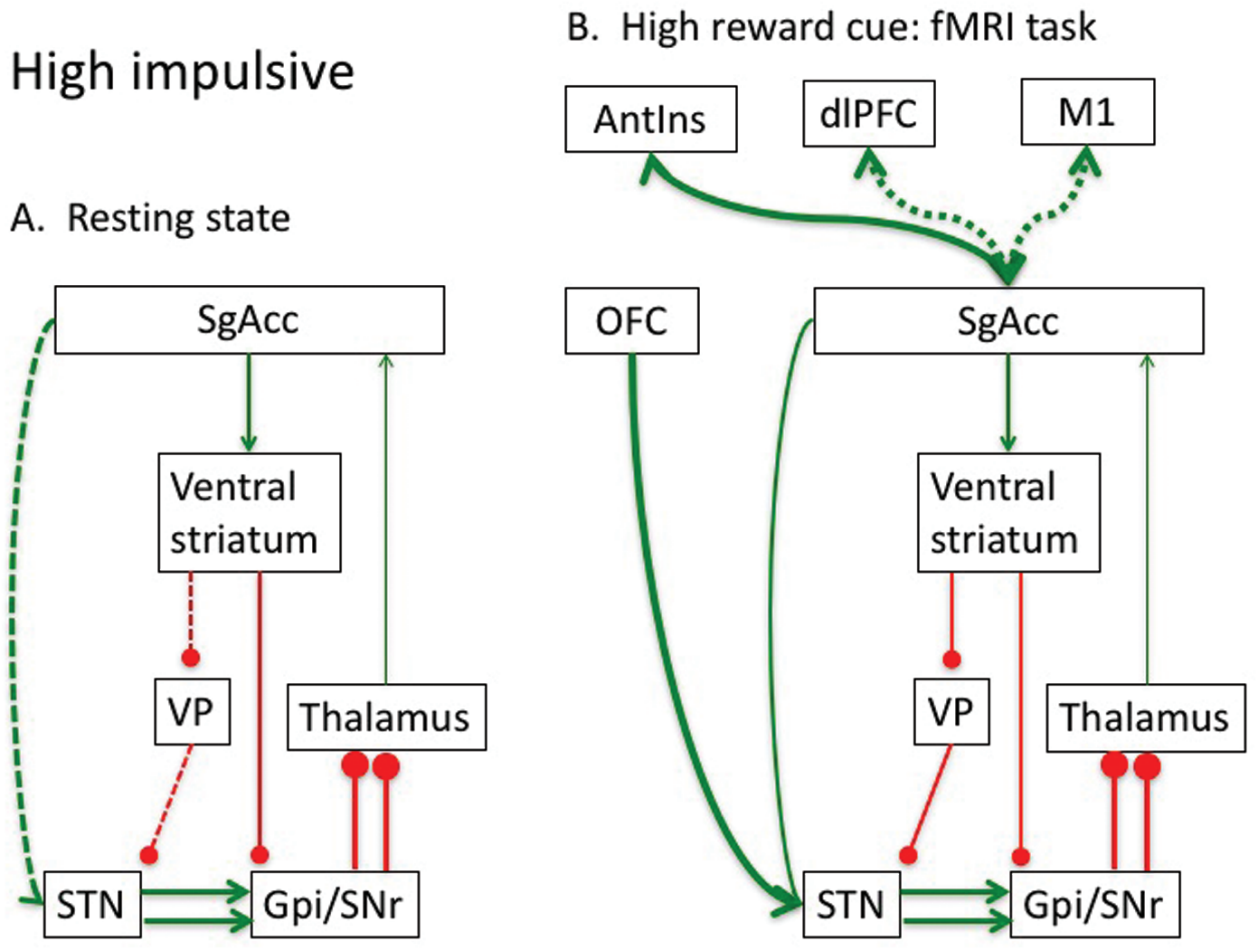
Waiting impulsivity model. (A) Waiting impulsivity at rest. Functional connectivity at rest suggests high impulsivity associated with decreased functional connectivity of indirect pathway implicating high tonic subthalamic nucleus (STN) output and impaired thalamocortical regulation. Decreased functional connectivity of the subgenual cingulate (SgAcc) and STN may be relevant to hyperdirect fast reactive signalling of environmental context. (B) Waiting impulsivity in response to high rewards. However, high impulsivity is associated with enhanced sensitivity to high value rewards with greater orbitofrontal cortex (OFC) activity and enhanced functional connectivity with the STN. In response to high reward cues, high impulsivity is associated with greater functional connectivity between the SgAcc, a region implicated in motivational processes, and the anterior insula (AntIns) implicated in the transition from impulsive to compulsive processes, and lower functional connectivity with regions implicated in response and motor control (dorsolateral prefrontal cortex, dlPFC; and M1).

Thus, at rest, high impulsivity is associated with decreased functional connectivity of the VS and STN (via the globus pallidus externa), thus disinhibiting STN output, shifting the balance of the indirect and direct pathways and decreasing thalamocortical regulation (Morris et al., 2016a) (Figure 3A). We have shown that SgAcc and STN resting state functional connectivity is decreased in high impulsivity, which may be most relevant for fast reactive signalling via the hyperdirect pathway. With exposure to high value reward cues, these current findings suggest that impulsivity is characterized by a shift towards engagement of regions implicated in subjective value related to choice and flexible behavior (OFC) and decreased engagement of regions implicated in higher order motor control (SMA). High impulsivity may be associated with enhanced sensitivity to the expectation of highly salient rewards and possibly a rapid OFC-STN signal of reward value influencing STN output and decreasing thalamo-cortical regulation (Figure 3B). The STN is believed to play a global modulatory role in impulse control and is critical for integrating contextual information (e.g., conflict) via hyperdirect pathways with action selection processes by modulating decision thresholds (Frank, 2006). More specifically, during high-conflict decisions, stimulation of the STN hastens anticipatory responding to high conflict decisions (Frank et al., 2007). We further show that in response to high reward cues, greater impulsivity is associated with enhanced SgAcc functional connectivity with the anterior insula but decreased with dlPFC and M1 (Figure 3B). In rodents, similar to lesions of the STN (Baunez and Robbins, 1997), lesions in the infralimbic cortex (equivalent to the human SgAcc) have been shown to increase premature responding (Chudasama et al., 2003). Motivational processes have been proposed as one of the possible mechanisms influencing waiting impulsivity. Evaluative motivational processes related to reward and punishment have been linked to altered SgACC functioning in maintaining dopaminergic-dependent reward activity (Pizzagalli et al., 2001). Furthermore, using a visual search paradigm that included a measure of motivational vigour, larger average rewards were linked to decreased activation in the SgAcc (Rigoli et al., 2016). Consistent with the role for SgAcc in behavioral inhibition, SgAcc activity to average rewards was linked to motor vigour. Enhanced SgAcc functional connectivity with the anterior insula is consistent with recent findings that the rodent anterior insula is implicated in waiting impulsivity with decreased cortical thickness and lesions enhancing waiting impulsivity. The anterior insula has also been suggested to play a critical role in the transition between impulsive towards compulsive behaviors (Belin-Rauscent et al., 2016). In contrast, regions implicated in action control including the SMA, and connectivity with regions involved in action selection and response and motor control including the dlPFC and motor regions, suggest decreased engagement of higher order response and motor control regions.

### Relationship with Other Forms of Impulsivity

In contrast to our findings, the relationship between the MID task and impulsivity indicates a consistent negative association between neural activity and self-reported impulsivity or delay discounting. For example, studies on disorders of alcohol users (Beck et al., 2009) or their unaffected children (Andrews et al., 2011) and gambling disorders (Balodis et al., 2012) showed that self-reported impulsivity correlated negatively with VS activity during response anticipation. Similarly, studies of ADHD showed decreased VS activity during the anticipatory phase, which also negatively correlated with self-reported impulsivity (Scheres et al., 2007). In healthy adults and healthy youths, both greater self-reported impulsivity and greater delay discounting were inversely related to VS (Vaidya et al., 2013) and left ventromedial caudate activity (Benningfield et al., 2014), respectively, during the response anticipation phase of the MID task. Adolescent smokers with greater delay discounting also showed lower VS activity during reward anticipation (Peters et al., 2011). However, our findings are highly compatible with a study demonstrating that greater trait reward sensitivity, measured using Gray’s impulsivity questionnaire, was positively correlated with VS and OFC activation for high magnitude reward anticipation (€1) but not for low magnitude anticipation (€0.50) (Hahn et al., 2009). These findings focusing on self-reported impulsivity or delay discounting predominantly report a negative relationship between neural activity in the response anticipation phase and impulsivity but a positive relationship with reward sensitivity. Our findings highlight that waiting impulsivity differs from self-reported impulsivity and delay discounting, thus emphasizing differences between subtypes of impulsivity and highlighting a relationship with enhanced reward sensitivity.

High waiting impulsivity has been associated with both sign-tracking and compulsive cocaine-seeking behaviors in rodents. Sign tracking rodents have enhanced approach behaviors towards the cue predicting reward (lever) rather than towards the location of food delivery, suggesting the cue has incentive properties (Davey and Cleland, 1982; Tomie et al., 1998). Following extinction, sign trackers are also more likely to show reinstatement of reward seeking following exposure to cocaine or food cues (Saunders and Robinson, 2010; Yager and Robinson, 2010). In contrast, goal trackers develop a similar behavior towards the location of food delivery itself rather than the cue (Flagel et al., 2009; Robinson et al., 2009). Sign tracking rodents with enhanced sensitivity to cues show greater premature responding as tested using a 2-choice serial reaction time task and a differential reinforcement of low rates of responding task (DRL). In the DRL task, rodents were first trained on a fixed reinforcement schedule 1 to learn to make an instrumental response for reward and subsequently trained on a DRL for 10 seconds and 20 seconds in which reinforcement occurs only if 10 or 20 seconds elapse between responses. These findings are specific to premature responding, as the sign tracking rodents do not show more impulsive choices or delay discounting (Lovic et al., 2011). Waiting impulsivity has also been shown to predict enhanced compulsive cocaine-seeking behaviors or lever presses despite receiving foot shocks (Belin et al., 2008). Our findings dovetail with preclinical reports of a relationship between waiting impulsivity and Pavlovian approach sign-tracking habits or instrumental habits perhaps mediated via enhanced reward sensitivity in those with high waiting impulsivity.

### Limitations and Conclusion

There were several limitations to this study. Firstly, the average number of premature responses in the 4-CSRT was rather low compared with scores in clinical populations. The 4-CSRT has previously been extensively described elsewhere and has been validated in alcohol- and methamphetamine-dependent subjects as well as recreational cannabis users and obese subjects with and without binge eating disorder. To enhance premature responding in healthy control subjects, the 4 test blocks with monetary feedback are optimized to increase premature responding. Optimization includes variability in target duration (block 2) and cue-target interval (block 3) and the presence of distractors (block 4). Secondly, the design of the MID task did not allow us to capture early responses made in the MID task. It would be informative to compare early responses in the MID task and premature responses outside the scanner in the 4-CSRT.

Our findings emphasize the relevance of reward sensitivity underlying waiting impulsivity. These findings differentiate waiting impulsivity from measures of anticipatory responding in motor tasks or conflict evaluation (Voon, 2014) and may have implications for the relationship between waiting impulsivity and incentive motivation and habit theories in addictions.

## Statement of Interest

None.
